# The relationship between heart rate and mortality of patients with acute coronary syndromes in the coronary intervention era: Meta-analysis

**DOI:** 10.1097/MD.0000000000005371

**Published:** 2016-11-18

**Authors:** Tan Xu, Youqin Zhan, Jianping Xiong, Nan Lu, Zhuoqiao He, Xi Su, Xuerui Tan

**Affiliations:** a Department of Cardiology, First Affiliated Hospital of Shantou University Medical, College, Shantou, Guangdong; b Department of Cardiology, Wuhan Asian Heart Hospital, Wuhan, Hubei, China.

**Keywords:** acute coronary syndrome, heart rate, percutaneous coronary intervention

## Abstract

**Background:**

Most of acute coronary syndromes (ACS) were receiving intervention treatment a high overall rate of coronary angiography in the modern medical practice.

Consequently, we conduct a review to determine the heart rate (HR) on the prognosis of ACS in the coronary intervention era.

**Methods:**

PubMed, EMBASE, MEDLINE, and the Cochrane Library was systematically searched up to May 2016 using the search terms “heart rate,” “acute coronary syndrome,” “acute myocardial infarction,” “ST elevation myocardial infarction,” “non-ST-segment elevation.” The outcome of interest was all-cause mortality. All analyses were performed using Review Manager.

**Results:**

Database searches retrieved 2324 citations. Eleven studies enrolling 156,374 patients were included. In-hospital mortality was significantly higher in the elevated HR group compared to the lower HR group (pooled RR 2.04, 95%CI 1.80–2.30, *P* < 0.0001). Individuals with elevated admission HR had increased risk of long-term mortality (Pooled RR = 1.63, 95%CI 1.27–2.10, *P* = 0.008) compared to lower admission HR. The pooled results showed elevated discharge and resting HR were related to increased mortality of patients with ACS (pooled RR 1.88, 95% CI 1.02–3.47, *P* = 0.04; pooled RR 2.14, 95%CI 1.37–3.33, *P* < 0.0001, respectively).

**Conclusion:**

Elevated HR may increase the mortality of ACS patients in the percutaneous coronary intervention era.

## 1 Introduction

More and more studies have revealed that the heart rate (HR) is a risk factor of mortality and cardiovascular morbidity in coronary artery disease, including patients with stable or acute coronary syndromes.^[1–7]^ Diaz et al^[1]^ has explored that patients suspected or proven coronary artery diseases (CAD) with resting HR > 83 bpm had a significantly higher risk for total mortality and cardiovascular mortality when compared with the HR ≤62 bpm group. In the modern era of primary percutaneous coronary intervention, an observational study^[2]^ has showed that hazard ratio for all-cause mortality in the elevated admission HR group (>70 bpm) was 1.59 for STEMI patients when compared with patients with an HR ≤70 bpm. Similarly, Antoni and his colleagues^[8]^ has concluded that patients with a discharge HR of ≥70 bpm had a 2 times increased risk of cardiovascular mortality at 1- and 4-year follow-up compared with patients with an HR < 70 bpm. However, another study^[6]^ prospectively enrolled 30,339 acute coronary syndromes (ACS) patients has demonstrated that admission HR >90 bpm or <50 bpm were associated with an increased risk of mortality. That means the relationship between HR and major adverse cardiac events followed a J-shaped curve with worst outcomes in the lowest and highest HR groups. Currently, some ACS risk models such as the PURSUIT^[9]^ and GRACE^[10]^ risk models have also included admission HR as a prognostic factor, modeled as a linear function. For example, in the GRACE risk model, the risk of events increased by 30% for every 30 beat increase in the heart rate (adjusted hazard ratio = 1.30; 95% CI = 1.23–1.47).

Inevitably, there calls into question the validity of a uniform “lower is better” paradigm or “J-shaped” between the heart rate and the prognosis of ACS patients in the contemporary practices. Most of ACS patients were receiving intervention treatment with a high overall rate of coronary angiography in the modern medical practice. Consequently, we conduct a systematic review and meta-analysis of clinical trials to determine the effect of HR on the prognosis of ACS patients in the coronary intervention era.

## 2 Material and methods

Our systematic review and meta-analysis were performed following the guidelines set forth in Preferred Reporting Items for Systematic Reviews and Meta-Analyses.^[11]^ And the ethical approval was not necessary because our meta-analysis was based on data from previously published studies.

### 2.1 Search strategy

**Figure 1:**
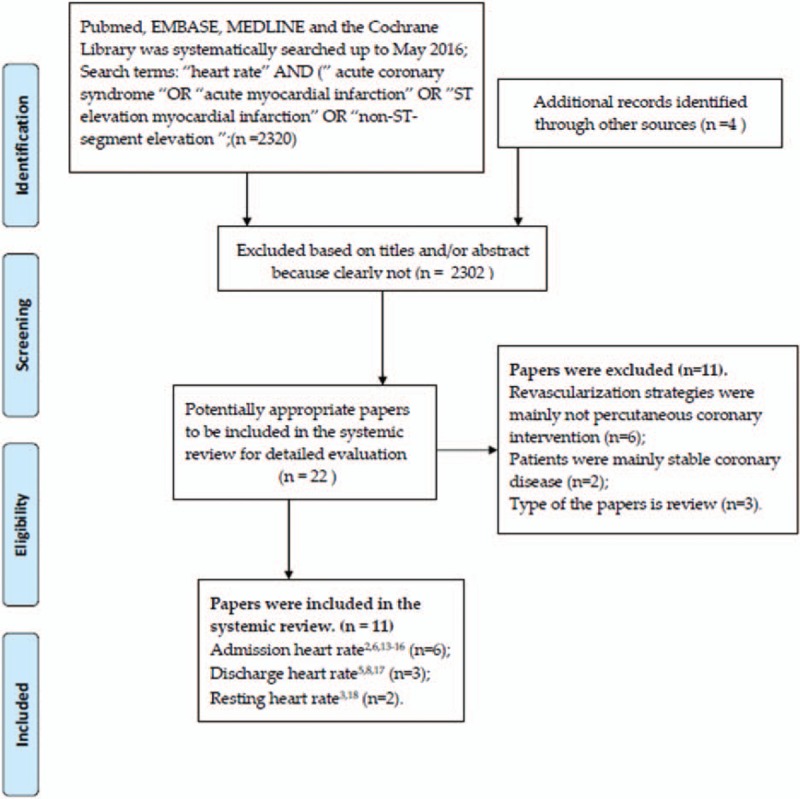
Flowchart of study selection.

PubMed, EMBASE, MEDLINE, and the Cochrane Library was systematically searched up to May 2016 using the search terms “heart rate,” “acute coronary syndrome,” “acute myocardial infarction,” “ST elevation myocardial infarction,” “non-ST-segment elevation” (see Fig. 1 for detailed search strategy). English language restriction was applied. The search was conducted by 2 independent researchers (TX and YZ).

### 2.2 Selection criteria

We included clinical trials to investigate the relationship between the heart rate and the prognosis of ACS. Eligible studies included adult patients with ACS including ST segment elevated myocardial infarction and non-ST segment elevated ACS treated with optimal percutaneous coronary intervention (PCI) and drug strategy. Studies were excluded if the reperfusion strategies were mainly coronary artery bypass grafting or fibrinolytic therapy.

Studies identified by the search strategy were screened by the title and abstract and excluded if they were not relevant to the research target by 2 investigators (TX and YZ). According to inclusion and exclusion criteria, the potentially eligible studies were then retrieved in the full text. When the eligibility of the studies exist divergence, a third investigator (XT) made the final decision. Citations of retrieved full text were also screened for eligible studies.

### 2.3 Data extraction and validity assessment

Data extraction was independently performed by 2 investigators (TX and YZ). Divergences were resolved by the aforementioned 2 reviewers’ consensus. Details of the publication (i.e., authors, year of publication), inclusion/exclusion criteria, and numbers of patients actually included in the analysis, demographics (patients’ age and gender), cardiovascular risk factors of the enrolled patients, percent of PCI strategies, and outcome definitions and events were collected and collated, mainly all-cause mortality.

The elevated HR included in the systemic review and meta-analysis, defined as the highest categories of HR, the other as lower HR. If the included studies have evaluated the adjusted confounding factors, the adjusted hazard ratio should be given priority to be extracted.

The outcomes of interest were (i) all-cause in-hospital mortality; (ii) long-term all-cause mortality. Endpoint definitions of the individual studies were included in the final analysis.

### 2.4 Statistical analysis

All the potentially cites of the systemic review and meta-analysis were managed by the EndNote software. All analyses were performed using Review Manager (RevMan version 5.3; Cochrane Collaboration, Oxford, UK). The unadjusted and multivariable-adjusted risk estimates for categorical (highest vs lower categories) outcome data (relative risks, hazard risks, and 95% confidence intervals) were transformed logarithmically in each study. The *I*^2^ statistic was used to test for heterogeneity, and the studies were pooled using fixed effects models with low heterogeneity (*I*^2^ < 50%).^[12]^ Otherwise, a random-effects model was used. Relative risks (RR) were used to pool outcomes with a 2-sided significance level of 5%. Individual trial and summary results are reported with 95% confidence intervals (CI). Sensitivity analysis was conducted to determine if an individual study was responsible for the observed effect by omitting 1 study and the risk of publication bias was assessed by examining the funnel plots. The statistical tests were 2-tailed with *P* < 0.05 chosen at the level of significance.

## 3 Results

Database searches retrieved 2324 citations. Most papers were excluded based on titles and/or abstract because clearly not relevant. Twenty-two potentially appropriate papers to be included for the full text review. According to the inclusion criteria, 11 studies enrolling 156,374 patients were eventually include in the systemic review. Six studies^[2,6,13–16]^ showed the relationship between admission HR and mortality of patients with ACS. Three studies^[5,8,17]^ measured the influence of discharge HR on the mortality of patients with ACS. The other 2 studies^[3,18]^ have demonstrated the relationship between resting HR and mortality of ACS patients.

### 3.1 Description of included studies and quality assessment

The baselines of study characteristics are summarized in Tables 1–3. Average age across all studies 65 years, 72.21% of patients were male. Patient follow-up ranged from 3 months to 5 years (median 23 months). The percentage of PCI strategies ranges from 44% to 100%. The revascularization strategy of 5 studies were total PCI.^[2,3,5,8,15]^ Three studies^[6,13,15]^ verified the J-shaped relationship between HR and mortality of patients with ACS. In despite of different HR levels, the aforementioned 3 studies all verified lower or elevated heart rate to be related to mortality of patients with ACS. The potential confounding adjusted factors differed across studies and the primary adjusted factors were age, sex, heart failure, and beta-blocker used.

**Table 1:**
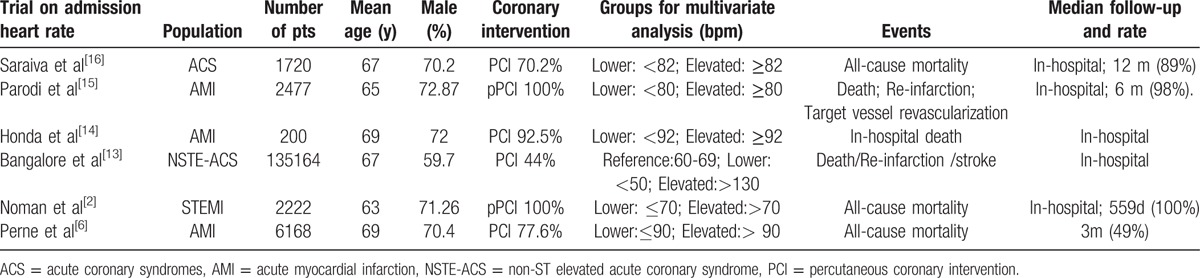
Study and participant summary characteristics on admission heart rate.

**Table 2:**
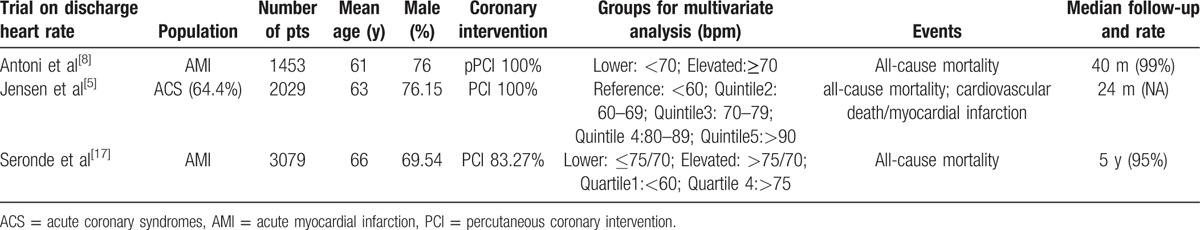
Study and participant summary characteristics on discharge heart rate.

**Table 3:**
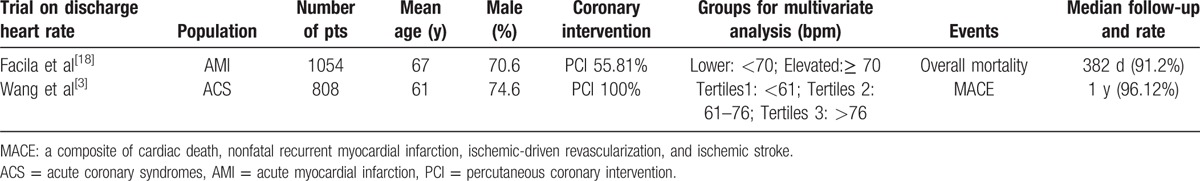
Study and participant summary characteristics on resting heart rate.

Study quality, where specified, was relatively high (Table 4). Based on the NOS quality assessment, 4 studies were defined as high quality (1 study scored 9 and 5 studies scored 7), and the other 5 studies were defined as moderate quality (3 studies scored 6 and 2 studies scored 5)

**Table 4:**
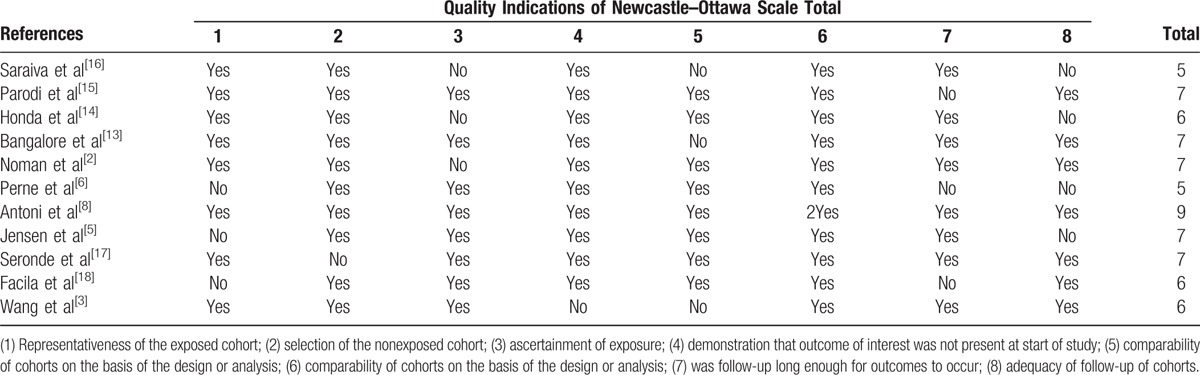
Assessment of study quality.

### 3.2 Quantitative data synthesis

#### 3.2.1 Admission heart rate and in-hospital mortality

**Figure 2:**
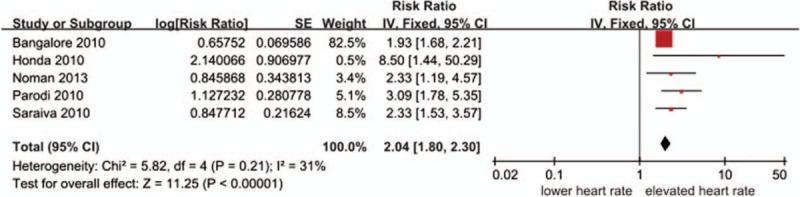
Relationship between the admission heart rate and in-hospital mortality.

All-cause in-hospital mortality were significant higher in the elevated HR group compared to the lower HR group (pooled RR 2.04, 95%CI 1.80–2.30, *P* < 0.0001; *I*^2^ = 31%) (Fig. 2). Unfortunately, the definition of elevated heart rate or lower admission heart rate is different. The dividing line mainly ranged from 70 bpm to 90 bpm. In the maximum weight study,^[13]^ the elevated heart rate was >130 bpm.

#### 3.2.2 Admission heart rate and long-term mortality

**Figure 3:**
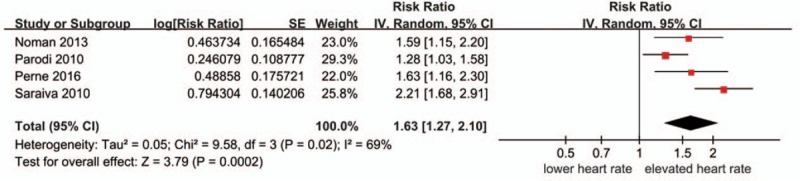
Relationship between the admission heart rate and long-term mortality.

This forest plot presents the association between elevated HR and long-term mortality compare to lower HR (Fig. 3). A statistical heterogeneity (*I*^2^ = 69%) was observed, so the random-effect model was used. The meta-analysis of the 4 studies suggested that individuals with elevated admission HR had an increased risk of long-term mortality (pooled RR = 1.63, 95%CI 1.27–2.10, *P* = 0.008) compared to lower admission HR. In this 4 included studies, the differences of elevated or lower heart rate are relatively minor.

#### 3.2.3 Discharge heart rate and long-term mortality

**Figure 4:**
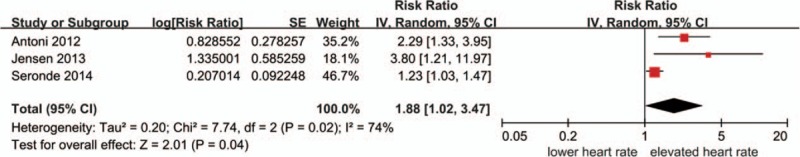
Relationship between discharge heart rate and long-term mortality.

Three included studies^[5,8,17]^ evaluated the relationship between discharge HR and long-term mortality. The follow-up duration ranged from 24 months to 5 years. The pooled results showed that elevated discharge HR was related to the increased mortality of patients with ACS (pooled RR 1.88, 95% CI 1.02–3.47, *P* = 0.04; *I*^2^ = 74%) (Fig. 4). Seronde et al^[17]^ presented different discharge HR categories and follow-up duration, ± 70 bpm as the dividing level for heart rate and 5 years follow-up morality were included in the meta-analysis. Similarly, the included follow-up duration of Antoni study^[8]^ was 4 years.

#### 3.2.4 Resting heart rate and long-term mortality or MACE

**Figure 5:**
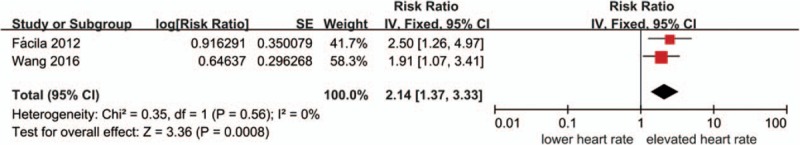
Relationship between resting heart rate and long-term mortality or MACE.

The definitions of resting HR were similar in the 2 included studies, between day 3 and 7 of the event once the patient was stable,^[18]^ or on 72 hours after onset of ACS during hospitalization.^[3]^ Elevated resting HR increased mortality or MACE of patients with ACS (pooled RR 2.14, 95%CI 1.37–3.33, *P* < 0.0001, *I*^2^ = 0%) (Fig. 5). MACE, major adverse cardiovascular events, includes a composite of cardiac death, nonfatal recurrent myocardial infarction, ischemic-driven revascularization, and ischemic stroke. The elevated heart rate of aforementioned 2 studies were similar, >76 bpm^[3]^ and ≥70 bpm,^[18]^ respectively. However, the events of interesting in the 2 included studies were not the same.

#### 3.2.5 Heterogeneity among included studies

Unfortunately, due to the differences in the definitions of elevated or lower HR, duration of follow-up, and the small number of events of some studies, we could not explore the sources of heterogeneity with subgroup analysis or meta-regression according to our prespecified procedures.

#### 3.2.6 Publication bias and funnel plots

**Figure 6:**
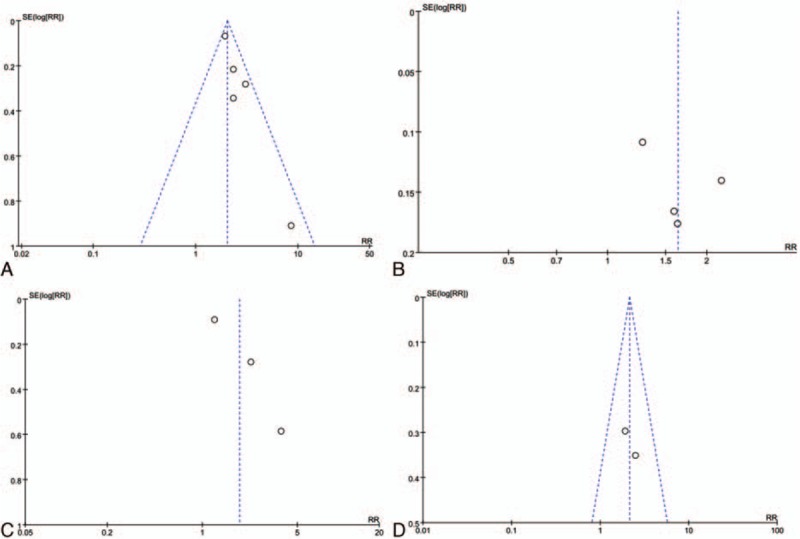
(A) The funnel plots of admission heart rate and in-hospital mortality. (B) The funnel plots of admission heart rate and long-term mortality. (C) The funnel plots of discharge heart rate and long-term mortality. (D) The funnel plots of resting heart rate and long-term mortality or MACE.

Owing to the small number of included studies about the influence of heart rate on mortality of patients with ACS in the era of PCI, with a maximum of 5 studies investigating admission heart rate and all-cause in-hospital mortality, the graphical or statistical assessment of publication bias was not sensitive. The funnel plots showed each comparison outcomes (Fig. 6A-D).

### 3.3 Sensitivity analyses

**Figure 7:**
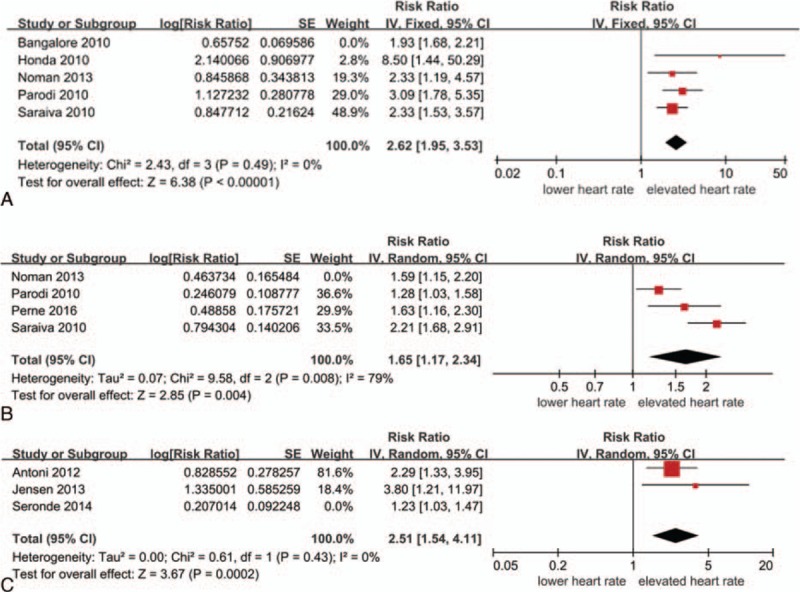
(A) The relationship between admission heart rate and in-hospital mortality by omitting 1 study. (B) The relationship between admission heart rate and long-term mortality by omitting 1 study. (C) The relationship between discharge heart rate and long-term mortality by omitting 1 study.

To analyze sensitivity, the primary results were not influenced by omitting 1 study except the resting heart rate and long-term mortality (Fig. 7A–C).

## 4 Discussion

In this systemic review and meta-analysis including 11studies in the era of PCI and >150,000 ACS patients, we demonstrated elevated HR is associated with a statistically significant increased risk of all-cause death of patients with ACS, in despite of admission, discharge or resting HR.

HR is increasingly been recognized as a modifiable risk factor for cardiovascular disease. In the contemporary practice, the PCI is the mainly revascularization strategies for coronary heart diseases, especially for the ACS patients. However, the prognostic significance of HR in a contemporary population undergoing PCI for ACS has not been systemically reviewed. Our systematic review and meta-analysis is the first to include 3 categories of heart rate together, admission, discharge, and resting HR, respectively.

For the sake of quantitative analysis, the relationship between heart rate and mortality of ACS patients, the HR was arbitrarily divided 2 parts, elevated or lower HR. The elevated admission heart rate was the predictor of ACS mortality in-hospital and long-term follow-up (pooled RR 2.04, 95% CI 1.80–2.30; pooled RR = 1.63, 95% CI 1.27–2.10, respectively). Similarly, the discharge and resting heart rate also increased the mortality of ACS patients (pooled RR 1.88, 95% CI 1.02–3.47; pooled RR 2.14, 95% CI 1.37–3.33, respectively).

In the systemic review, 3 studies^[6,13,15]^ showed the J-shaped relationship between heart rate and mortality of patients with ACS. Bangalore et al^[13]^ has been evaluated that the relationship between admission heart rate and mortality followed a “J-shaped” curve, <50 bpm or >130 bpm as increased mortality compared with 60 to 69 bpm. As suggested by J-shaped relationship, we should paid attention to the extreme conditions when explaining the lower or elevated heart rate.

A meta-regression of randomized clinical trials^[19]^ has verified quantitative relationship between resting heart rate reduction and magnitude of clinical benefits in post-myocardial infarction. A statistically significant relationship was found between resting HR reduction and the clinical benefit including reduction in cardiac death, all-cause death, sudden death, and non-fatal myocardial infarction recurrence. Each 10 bpm reduction in the HR is estimated to reduce the relative risk of cardiac death by 30%. This meta-analysis indirectly showed the hazard of elevated HR for myocardial infarction patients. However, the included studies were all pre-PCI era.

The pathophysiological mechanism of HR-related mortality is still elusive. It has been demonstrated that in patients with CAD, elevated HR produces coronary vasoconstriction, potentially further impairing oxygen supply.^[20]^ Other study^[21]^ has shown that an elevated HR might influence the atherosclerotic coronary disease progression and plaque stability. Inevitably, HR is regarded as a phenomenon, as it derives from the depolarization rate of the sinoatrial node that in its turn largely derives from the activity of the autonomic nervous system. Thus, HR is directly related to sympathetic activity or autonomic imbalance. It is unknown whether heart rate mediates the deleterious effects of sympathetic hyperactivity or contribute per se to patient outcome.^[15]^

The optimal admission, discharge, and resting HR were unable to be given for clinical practice because there was not consistent definition of elevated or lower HR in the included studies. However, most of the included studies have regarded >70 to 80 bpm as the elevated HR.^[2,3,8,15–18]^ Therefore, >70 to 80 bpm should be identified as the risk factor for mortality of ACS patients.

## 5 Study limitations

The present study must be interpreted within the context of its potential limitations. First, heterogeneity: There was significant heterogeneity among the included studies for the analysis relationship between admission or discharge HR and long-term mortality. However, as a result of the limited number of included studies for each outcome, we could not identify the sources of heterogeneity. Second, the included studies have not the same criteria for elevate or lower HR, which may have substantial detrimental on the explanation of the pooled results. Thus, the optimal HR for clinical practice on ACS patients cannot be given. Third, although revascularization strategy of 5 studies was total PCI, yet strategy in some included studies were not totally PCI, which may also influence the pooled results on the behalf of PCI era.

## 6 Conclusion

Our systematic review reveals that elevated admission, discharge, and resting HR may increase the mortality of ACS patients in the PCI era. As J-shaped relationship existed, the extreme conditions should be paid attention when explaining the lower or elevated heart rate. Because there are not identical definition of elevated or lower HR, there need large cohort studies to confirm optimal heart rate for clinical practice in the future.
